# Diagnosis of frailty in geriatric patients: Is the pictorial fit frail scale an appropriate screening tool in hospital settings?

**DOI:** 10.22088/cjim.12.3.307

**Published:** 2021-04

**Authors:** Maryam Chehrehgosha, Mahtab Alizadeh-Khoei, Nasser Behnampour, Farshad Sharifi, Reza Fadaye Vatan, Reyhaneh Aminalroaya

**Affiliations:** 1 1.Department of Gerontology, University of Social Welfare and Rehabilitation Sciences, Tehran, Iran; 2 2.Department of Surgical Technology, Paramedical School, Golestan University of Medical Sciences, Gorgan, Iran; 3 3.Department of Clinical Gerontology & Geriatric, Medical School, Tehran University of Medical Sciences, Tehran, Iran; 4 4.Department of Biostatistics and Epidemiology, Health Management and Social Development Research Center, Golestan University of Medical Sciences, Gorgan, Iran; 5 5.Elderly Health Research Center, Endocrinology and Metabolism Population Sciences Institute, Tehran University of Medical Sciences, Tehran, Iran; 6 Iranian Research Center of Aging, University of Social Welfare and Rehabilitation Sciences, Tehran, Iran; 7 Department of Geriatric, Ziaiyan Hospital, Medical School, Tehran University of Medical Sciences, Tehran, Iran

**Keywords:** Frailty, Pictorial Fit Frail Scale, MDS-specific frailty index, Hospital, ROC Curve

## Abstract

**Background:**

Frailty is accompanied by serious health complications in the elderly, especially during hospitalization. Visual scales have been designed for quick and easy evaluation of frailty in different cultures and settings. Therefore, this study aimed to define the accuracy of the Pictorial Fit Frail Scale (PFFS) for frailty screening in the hospitalized elderly in Iran.

**Methods:**

This cross-sectional study was conducted on 304 hospitalized participants, aged 65-85 years old admitted at Ziaeian Hospital (Tehran) were enrolled through the inclusion criteria from August to December 2019. All participants were evaluated based on the Minimum Data Set-Home Care, the Pictorial Fit Frail Scale, and the Quality-of-Life instrument, through face-to-face interviews by a trained nurse at the admission time. Spearman’s correlation coefficient, and ROC analysis were performed using SPSS at p<0.05.

**Results:**

The highest correlation was obtained by Frailty Index (FI) and PFFS (0.770). FI had a negative correlation with QoL (-0.48). The optimal cut-points for PFFS according to FI ≤ 0.08 (robust vs. pre-frail) was obtained 0.10 with sensitivity, specificity, positive predictive value (PPV), negative predictive value (NPV), and accuracy 100.00% and the best cut-point for PFFS based on FI ≥ 0.25 (pre-frail vs. frail) was obtained 0.29 with sensitivity and specificity, positive predictive value (PPV), negative predictive value (NPV), and accuracy 100.00%.

**Conclusion:**

It seems the Pictorial Fit-Frail Scale (PFFS) is a reliable scale with a high level of accuracy, and excellent sensitivity and specificity to measure the frailty level in hospitalized elderlies.

Frailty is “a syndrome caused by the declined functional capacity of several body systems to such an extent that the body's physiological function is close to or on the verge of clinical failure". The outcomes of the frailty in the elderly include higher risks of disability, functional decline, fall, hospital re-admission, and death (1,2), which may compromise their quality of life (3). The frailty prevalence is varied based on the different diagnosis criteria, tools, and settings, and reported between 2.3% and 75.0% in the different studies in community dueling population (4,5). The frail elderly are more likely to be hospitalized with an acute illness, and their hospitalization stay is longer than robust patients (6).

The frail elderly are more likely to be hospitalized with an acute illness, and their hospitalization stay is longer than robust patients (6). The prevalence of frailty in hospital settings range between 10.4 and 37.0% (7).  Hence, the elderly screening in a clinical setting can help detect pre-frail and high-risk frail patients for negative health outcomes (8,9). 

There are different tools for assessing frailty in the elderly. It is still unclear which tools are more appropriate for screening frail elderly patients in clinical settings (10). The Frailty index (cumulative deficit model) is suitable for evaluating hospitalized patients because a large number of health-related variables are usually applied (11). The FI can be obtained from the Comprehensive Geriatric Assessment (CGA) or the Minimum Data- Home Care (MDS-HC) (12, 13). Based on CGA and MDS, the FI is considered the gold standard for diagnosing frailty in some studies (14, 15); however, it is often not practical due to the long implementation time and a large number of evaluations (16). Several methods, instruments, and questionnaires have been developed for frailty assessment. However, there is no prevailing consensus over the definition of frailty for clinical uses, such as screening and diagnosis (17, 18). This is because the majority of them are time-consuming and costly, and require specific assessment tools and experienced specialists (19).

The Pictorial Fit Frail Scale (PFFS) is a visual scale that was developed by Theou et al. (2019) for frailty assessment (20). It uses visual images to assess frailty in different cultures and different levels of cognitive patients (21). As compared to its counterparts, PFFS uses a simpler, easier, and more practical and sensitive tool to measure the frailty level. As an advantage, this tool is not under the influence of cultural differences and literacy levels of the patients (22). The content validity, feasibility, and inter-rater reliability of the scale were examined by the different studies (20,22). 

The correlation between the PFFS and the CGA-based frailty index was reported relatively high (22). This study aimed to define the accuracy of the Pictorial Fit Frail Scale (PFFS) for frailty screening in the hospitalized elderly in accordance with the MDS-HC, considered the gold standard. 

## Methods

**Study design:** This cross-sectional study was conducted on the hospitalized elderly who were admitted at a teaching hospital affiliated with Tehran University of Medical Sciences (Ziaeian Hospital) from August-December, 2019. 

**Inclusion and exclusion criteria:** The patients entered the study with the following criteria: (1) hospitalized elderly aged 65-85 years, (2) not being admitted into the ICU, (3) the elderly being no residents in a nursing home, (4) being accompanied by an informed caregiver to obtain the consent and information in the elderly without mental capacity or in cases of severe illness. The elders who were unable to respond to more than 40% of the questions or had a severe problem were excluded. After admitting the elderly to the hospital and obtaining informed consent from the participants and their companions, their health-related information was collected and frailty assessment was performed through in-person interviews by a nurse in the research team. Data collection was done by completing relevant forms in the presence of the elderly and their caregivers.

**Data Collection: ADL and IADL: **ADL evaluates the mobility, transfer, locomotion, dressing, eating, toilet use, and personal hygiene of the elderly and IADL contains meal preparation, ordinary housework, financial management, medication management, phone use, shopping, and transportation domains (23). Dependence in each domain of ADL and IADL was considered an impairment. ADL and IADL scores are obtained from the summation of all domains (24). ADL and IADL were evaluated using the MDS-HC form. The MDS-HC has been validated by Mehdipour et al.in Iran (25).

**Quality of Life: **Quality of life was assessed by the EQ5D-3L, which evaluates mobility, self-care, usual activities, pain, and anxiety. The total score of 5 dimensions translates to the "index score" by the interim mapping method. The highest and the lowest index scores were 1.00 and -0.769, respectively (26). The reliability (intra class correlation coefficients) of the EQ-5D Index has been reported 0.753 (27). 

**Frailty Assessment:**

- **MDS-Specific Frailty Index: **The MDS-HC is a geriatric standard assessment that includes more than 200 items and is used for individual care plans(28). The FI was developed by using 42 health-related variables derived from the selected domain of the MDS-HC form(29). Each variable was re-coded on 0, 0.5, or 1 scores. The frailty index was calculated by summing all scores divided by the total score of possible defects. It ranges between 0 to 1(29,30). More information was published in a study by Burn et al.(31) The MDS-HC has been validated by Mehdipour et al. in Iran (25). Based on a study by Ge et al. (2019), the FI cut-point include robust (≤0.08), pre-frail (0.08 <FI< 0.25), and frail (≥0.25) cases(32)^.^
- **Pictorial Fit Frail Scale (PFFS): **The PFFS consists of 14 domains including mood, medication use, mobility, function, balance, social communication, daytime tiredness, memory and thinking, vision, hearing, pain, history of weight loss, aggression, and bladder control. This scale represents the usual states of the elderly (20). The optimal and worse levels are defined for each level. The score of each domain varies from 0 to a maximum of 6. Ranging from 0 to 43, the total raw scores were calculated by summing all domains. The standardized score was calculated by dividing the raw score to the total possible score (43), which ranged between 0 and 1. The closer the score to zero, the better the conditions of the elderly (22). The content validity, feasibility, and inter-rater reliability of the scale have been measured in different studies (20,22).

**Background Variables: **Demographic information and patient-related factors were collected (age, sex, marital status, living arrangements, comorbidity, polypharmacy). The co-existence of at least 3 chronic illnesses was defined as comorbidity (33), and concurrent use of five or more medications was defined as polypharmacy (34). 

**Statistical Analysis: **The statistical analysis was performed in SPSS 18.0 for Windows (SPSS, Inc., Chicago, IL, USA), at p-values <0.05. Continuous and categorical variables were presented as the mean (SD) or numbers and proportions, respectively. The correlation coefficient was employed to assess the relationships of the PFFS with the FI, ADL, IADL, and QOL. In the ROC analysis for detecting binary cut-points for the PFFS, the three FI subgroups were integrated into the two subgroups: (FI≤0.08; robust vs. pre-frail+ frail) and (FI≥ 0.25; robust +pre-frail vs. frail) (32).

 For the precise detection of robust, pre-frail, and frail status in the elderly patients, we omitted the frail patients in FI≤ 0.08 (robust vs. pre-frail) and also omitted the robust patients in FI≥ 0.25 (pre-frail vs. frail). The diagnostic accuracy was evaluated in terms of sensitivity, specificity, positive predictive value (PPV), negative predictive value (NPV), and positive likelihood ratio (PLR), negative likelihood ratio (NLR), and accuracy of 95% CI.

**Ethics:** Informed consent was gained from the patients or their legal representatives. The Ethics Committee of the University of Social Welfare and Rehabilitation approved the project under the Code: IR.USWR.REC.1396.296.

## Results

A total of 304 hospitalized elderly patients participated in this study. The mean age of the participants was 75.71 ± 6.29, and 74% of them were women. More than half of the elderly patients were widows (53.61%). Moreover, 87.18% of them had polypharmacy, and 51% experienced more than 3 comorbidities. The mean (SD) of QOL was 0.57±0.31 (table 1). 

**Table1:** Patient's characteristics

**Variables**		**[Mean ± SD, or n (%)]**
Age		75.71 ± 6.29
Gender	Male	79 (26%)
	Female	225 (74%)
Marital status	Single	2 (0.65%)
	Married	137 (45.06%)
	Widow	163 (53.61%)
	Divorced	2 (0.65%)
Living arrangement	Alone	70 (20.3%)
	With husband	108 (35.5%)
	With husband and child	29 (9.53%)
	With child	76 (25%)
	With other	21 (6.9%)
Comorbidity	3< Disease	149 (49%)
	3≥ Disease	155 (51%)
Polypharmacy	5< Drug	39 (12.82%)
	5≥ Drug	265 (87.18%)
ADL		5.03 ± 7.20
IADL		11.65 ± 5.28
QOL		0.57 ± 0.31
MDS-specific frailty index		0.21 ± 0.007
FI-MDS. Category	Robuts	3 (1.00%)
	Pre-frail	222 (73.00%)
	Frail	79 (26.00%)
Pictorial Fit Frail Scale (PFFS)		0.33 ± 0.026

The PFFS had no significant difference from comorbidity and polypharmacy (P>0.05). Table 2 presents the correlations of PFFS scores with FI, ADL, IADL and QOL. The PFFS had a higher correlation with the FI (r=0.77); moreover, the lowest correlation was observed with QOL (r=-0.48).

**Table 2:** Correlation between the PFFS and FI, ADL, IADL and QOL

**Measurement**	**r (95% CI)**	**P-Value**
FI	0.77 (0.76 – 0.771)	< 0.001
ADL	0.59 (0.51 – 0.66)	< 0.001
IADL	(0.49 – 0.64)	< 0.001
QOL	-0.48 (-0.39 – -0.57)	< 0.001

The ROC analysis results for binary cut-points of the PFFS, the results showed that the cut-point for the PFFS in FI≤ 0.08 (robust vs. pre-frail +frail) was 0.10 with a sensitivity of 97.67% and a specificity of 66.67%. The optimal cut-point for the PFFS in FI≥ 0.25 (robust +pre-frail vs. frail) was 0.31 with a sensitivity of 91.11% and a specificity of 71.96% (table 3). In the precise detection of robust patients from pre-frail patients (frail patients were omitted), the results showed that the optimal cut-point for the PFFS in FI≤ 0.08 was 0.104 with sensitivity and specificity of 100%. Moreover, in differentiating pre-frail from frail patients (robust patients was omitted), the optimal cut-point for the PFFS in FI≥ 0.25 was 0.290 with sensitivity and specificity of 100% (table 4).

**Figure 1:**
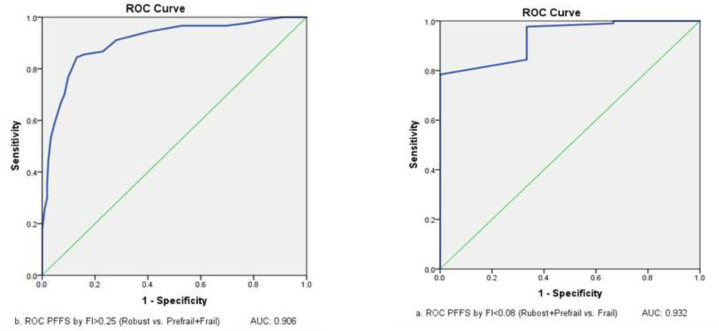
ROC curves of PFFS with FI≤ 0.08 (robust vs. pre-frail +frail) and FI≥ 0.25 (robust +pre-frail vs. frail)

**Figure 2:**
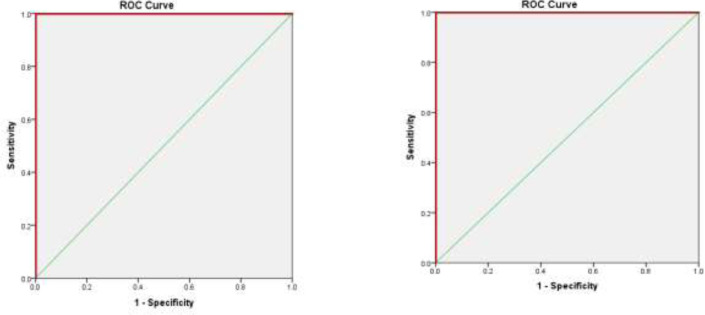
ROC curves of PFFS with FI≤ 0.08 (robust vs. pre-frail) and FI≥ 0.25 (pre-frail vs. frail)

Figures 1 and 2 shows the AUC of PFFS by FI ≤0.08 and FI≥0.25 in two different categories.

**Table 3:** The Sensitivity and specificity of PFFS for binary classification

**Criteria** **(MDS-specific frailty index)**	**PFFS** **Cut point**	**Sensitivity** **(95% CI)**	**Specificity** **(95% CI)**	**PPV** **(95% CI)**	**NPV** **(95% CI)**	**PLR** **(95% CI)**	**NLR** **(95% CI)**	**Accuracy** **(95% CI)**	**AUC** **(95% CI)**
FI ≤ 0.08 (Robust vs. Pre-frail + Frail)	0.10	97.67% (95.27- 99.06)	66.67% (9.43- 99.16)	99.66% (98.34- 99.93)	22.22% (8.81- 45.81)	2.93 (0.59-14.52)	0.034 (0.01-0.10)	97.37% (94.88- 98.86)	0.932 (0.835- 1000)
FI ≥ 0.25 (Robust + Pre-frail vs. Frail)	0.31	91.11% (83.23- 96.08)	71.96% (65.43- 77.87)	57.75% (52.20- 63.10)	95.06% (90.81- 97.40)	3.25 (2.60-4.07)	0.12 (0.06-0.24)	77.63% (72.52- 82.19)	0.906 (0.866- 0.945)

PPV: Positive Predictive Value

NPV: Negative Predictive Value

PLR: Positive Likelihood Ratio

NLR: Negative Likelihood Ratio

AUC: Area Under Curve

**Table 4:** The sensitivity and specificity of the PFFS for precise detection of robust, pre-frail, and frail status

**Criteria** **MDS-specific frailty index**	**PFFS** **Cut point**	**Sensitivity** **(95% CI)**	**Specificity** **(95% CI)**	**PPV** **(95% CI)**	**NPV** **(95% CI)**	**PLR** **(95% CI)**	**NLR** **(95% CI)**	**Accuracy** **(95% CI)**	**AUC** **(95% CI)**
FI ≤ 0.08 (Robust vs. Pre-frail)	0.10	100.00% (66.37-100.00)	100.00% (97.45-100.00)	100.00	100.00	-	0.00	100.00% (97.60- 100.00)	1.00 (1.00- 1.00)
FI ≥ 0.25 (Pre-frail vs. Frail)	0.29	100.00% (97.45-100.00)	100.00% (97.60-100.00)	100.00	100.00	-	0.00	100.00% (97.60- 100.00)	1.00 (98.76- 1.00)

PPV: Positive Predictive Value, NPV: Negative Predictive Value, PLR: Positive Likelihood Ratio, NLR: Negative Likelihood Ratio, AUC: Area under Curve

a. ROC PFFS by FI<0.08 (Robust vs. Pre-frail), AUC: 1.000 b. ROC PFFS by FI>0.25 (Pre-frail vs. Frail), AUC: 1.000

## Discussion

The study findings showed that the PFFS had a high correlation with the FI, an excellent AUC, and highest sensitivity and specificity (100%) in two cut points of 0.1 and 0.29. The PFFS has attracted a great deal of attention because it can be used in a variety of clinical settings, designed on short and concise visual scales, and considered an alternative to frailty assessment based on a comprehensive geriatric assessment (20). Some studies reported that the evaluation based on the phenotype model seemed to help diagnose pre-frail patients so that preventive measures could be taken for this group of the elderly. Frailty indices also act better in predicting mortality and long-term outcomes (35, 36); moreover, the FI has a higher predictive power to detect adverse clinical events than other tools in hospital and community settings (37–39). Although the PFFS is a visual frailty scale, the result show that it can come in handy in hospital settings and help detect the frailty status. According to the findings of this study, there was a high correlation between the PFFS and the FI. In line with our results, Wallace et al. (2019) reported that there was a moderately high correlation between the PFFS and the FI-CGA among the elderly in memory clinics (22). In consistence with a study by Vanleerberghe et al. (2019), there was an inverse correlation between the Comprehensive Frailty Assessment Instrument (CFAI) and QOL (40). Similar to a study by Gregorevic et al. (2016), the results indicated that deterioration in frailty correlated with functional decline(41). A meta-analysis showed that the frailty level could increase the limitation of daily living activities, physical disability, recurrent falls, hospitalization, and mortality (42). Frailty can result in gait speed and muscle strength decline and is often associated with other health problems (43), such as functional dependency, ADL, and IADL. On the other hand, the frailty-caused functional disability, dependency, and psychological pattern changes could impair the autonomy and well-being of the elderly (44,45). As a result, frailty can reduce QoL (40) and is negatively correlated with it.

In a systematic review study by de Vries et al., eight essential components were proposed for the ideal frailty measures as nutrition, mobility, physical activity, resistance, energy, mood, cognition, and social connections (46). Apparently, the PFFS contains the important geriatric domains; however, these domains have been minimized and can be assessed visually for easier administration (22). Therefore, considerations of these domains for the development of the PFFS can probably justify the correlation of the PFFS with the FI, ADL, IADL, and QOL.

The results of this study also showed that the AUC in two cut-point categories of the FI was excellent and had a good sensitivity in different cut-points. In a study by Jung et al. (2020), the AUC for the Korean FI based on the CHS frailty scale was reported 0.82 with a sensitivity of 81.6% (47). In another study, the AUC for the Korean FI–Primary care based on Fried phenotype criteria was reported 0.921 with a sensitivity of 89% (48).The results showed that the PFFS, such as the Frailty index, is effective in measuring the frailty level in the elderly (22). The analysis of MDS-HC and PFFS was done by a trained nurse. To measure the PFFS accuracy in distinguishing the pre-frail people from frail patients, the healthy individuals were excluded from the analysis; moreover, to distinguish the healthy people from pre-frail ones, the elderly with frailty were removed from the analysis. Therefore, it seems that the aforementioned factors have an influence on the operating characteristic curve (AUC) of this scale. There have been no studies setting cut-points for the PFFS and conducing an ROC analysis. The existing papers only mention the agreement between different assessors and content validity. For instance, Theou et al. (2019) evaluated only face and content validities. They reported a 66% agreement with feasibility and usefulness of the PFFS (20). The goal of the PFFS is to create a simple, easy-to-use screening tool to which cultural complications do not pose a problem. This tool should also be used easily by the elderly with visual impairment and should not need any experts. It should be used in any settings (21).

Appropriate results have been reported in the few published studies evaluating this tool, and promising findings have been obtained in hospital settings in this study. However, more evaluations should be performed by different evaluators in different settings.

## Strengths:

The strength of this study was that most of the participants were illiterate, although we could detect a strong validation for the FPPS. In other words, this study could be easily applied to the illiterate elderly that might account for a major proportion of the elderly in developing countries.

## Limitations:

The major limitation of the study was the lack of a second assessment after the patients were discharged from the hospital. Apparently, the PFFS is a suitable scale for detecting frailty in the hospitalized elderly similar to the FI. It can be employed to evaluate frailty at short notice with the equal precision of the existing gold standard tools. Moreover, the functional status and quality of life of the hospitalized elderly should be taken into account along with the assessment of their frailty conditions because changes in these components can be involved in the outcomes of frailty.
